# *In vivo* imaging of prostate cancer using an anti-PSMA scFv fragment as a probe

**DOI:** 10.1038/srep23314

**Published:** 2016-03-21

**Authors:** Claire Mazzocco, Giulio Fracasso, Coralie Germain-Genevois, Nathalie Dugot-Senant, Mariangela Figini, Marco Colombatti, Nicolas Grenier, Franck Couillaud

**Affiliations:** 1 CNRS UMS 3428 and Univ. Bordeaux, 146 rue Léo Saignat, F33076 Bordeaux; 2 Department of Medicine, Verona University, Italy; 3 Service d’Histologie INSERM US005, Univ. Bordeaux, 146 rue Léo Saignat, F33076 Bordeaux; 4 Molecular Therapies Unit, Department of Experimental Oncology and Molecular Medicine, Fondazione IRCCS Instituto Nazionale dei Tumori, Milano, Italy; 5 Service d’Imagerie Diagnostique et Interventionnelle de l’Adulte, Groupe Hospitalier Pellegrin, Place Amélie Raba-Léon – F 33076 BORDEAUX Cedex; 6 Univ. Bordeaux, Imagerie Moléculaire et Thérapies Innovantes en Oncologie (IMOTION), 146 rue Léo Saignat, F33076 Bordeaux

## Abstract

We aimed to evaluate a fluorescent-labeled single chain variable fragment (scFv) of the anti-PSMA antibody as a specific probe for the detection of prostate cancer by *in vivo* fluorescence imaging. An orthotopic model of prostate cancer was generated by injecting LNCaP cells into the prostate lobe. ScFvD2B, a high affinity anti-PSMA antibody fragment, was labeled using a near-infrared fluorophore to generate a specific imaging probe (X770-scFvD2B). PSMA-unrelated scFv-X770 was used as a control. Probes were injected intravenously into mice with prostate tumors and fluorescence was monitored *in vivo* by fluorescence molecular tomography (FMT). *In vitro* assays showed that X770-scFvD2B specifically bound to PSMA and was internalized in PSMA-expressing LNCaP cells. After intravenous injection, X770-scFvD2B was detected *in vivo* by FMT in the prostate region. On excised prostates the scFv probe co-localized with the cancer cells and was found in PSMA-expressing cells. The PSMA-unrelated scFv used as a control did not label the prostate cancer cells. Our data demonstrate that scFvD2B is a high affinity contrast agent for *in vivo* detection of PSMA-expressing cells in the prostate. NIR-labeled scFvD2B could thus be further developed as a clinical probe for imaging-guided targeted biopsies.

Prostatic carcinoma (PCa) is the most common cancer in men^1^ and the second cause of cancer-related deaths for North American and European men. Its aggressiveness depends on the extent of the tumor and the Gleason score which ranges from 2 (slow evolution) to 10 (fast evolution). Early detection can greatly enhance life expectancy (with survival rates reaching 100%), whereas survival rates rapidly decrease if the tumor spreads over the prostate gland^2^.

The current PCa diagnostic strategy which combines digital rectal examination and blood prostate-specific antigen (PSA) screening followed by transrectal guided biopsies may reduce specific mortality, but this comes at the cost of overdiagnosis and overtreatment of indolent tumors^3^. As such, the clinical benefit of this diagnostic work-up is uncertain^4^. In clinical practice, the initial biopsy scheme is based on blind sampling of 10–14 cores; this procedure shows a relatively low overall cancer detection rate of 27–40.3%^5^,^6^,^7^. More specific imaging tools to improve the detection rate of significant tumors with biopsies are thus necessary to improve the management of PCa.

Multiparametric magnetic resonance (MR) imaging followed by targeted biopsies has already been demonstrated to improve PCa diagnosis with a decreased detection rate of low-risk PCa and an increased detection rate of intermediate/high-risk PCa^8^. However, multiparametric MR imaging lacks specificity and the time required for large-scale detection of PCa makes this strategy difficult to implement due to MR scanner accessibility. Moreover, subsequent image-guided targeted biopsies still require sophisticated fusion systems or costly MR-guidance. The development of new specific molecular imaging strategies is required to accurately identify significant prostatic tumors and to guide biopsies of malignant lesions.

Currently, the prostate specific membrane antigen (PSMA), a type II transmembrane protein produced by the prostatic epithelium, is one of the promising molecular targets for PCa detection^9^. It is a prominent prostate cancer marker due to its over-expression in all stages of the disease, from primary to metastatic^9^,^10^,^11^. PSMA is also expressed in several healthy tissues like the prostatic epithelium, kidney, small intestine and salivary glands, but its expression rate is increased from 100- to 1,000-fold in PCa^10^. Furthermore, the ability of PSMA to be internalized after ligand binding is an attractive property that may improve efficacy for diagnostic and therapeutic purposes^12^,^13^,^14^.

PSMA has already been approved as a diagnostic target for scintigraphy with ProstaScint®, the radio-labeled murine monoclonal antibody (mAb) 7E11. This mAb recognizes an internal epitope of PSMA not accessible to circulating antibodies and therefore staining necrotic cells only^15^,^16^. More recently, the humanized J591monoclonal antibody targeting the extracellular epitope of PSMA available on live and dead cells was proposed and provided significant benefits in PCa imaging, leading to clinical trials in advanced cancer. In addition to antibodies, several small molecules have been described as ligands to the extracellular domain of PSMA, such as DCFBC or HBED-CC^17^,^18^. Using these ligands with ^68^Gallium- or ^18^Fluor-labeling, there has been a rapid development for PSMA-PET in metastatic or recurrent prostate cancer imaging in several European countries^19^. Recently, together with a combination of morphological and multiparametric functional information, PET/MRI technology was demonstrated to offer better detection of prostate cancer^20^, but its low availability and high cost preclude its use in large detection strategies and primary biopsy guidance.

Whereas whole antibodies also produced nonspecific binding due to the engagement of Fc receptors^21^, antibody fragments such as single chain variable fragments (scFv) of antibodies and minibodies seemed to be even more specific, hence the growing interest for their use as immuno-imaging probes targeting cancer cells^22^. Alternatively to antibody-based tools, PSMA inhibitors and aptamer to PSMA^23^ have also been proposed as efficient targeting agents for PCa.

Given the limitations of other imaging modalities to identify prostatic carcinoma on a large scale and to guide targeted biopsies, a hybrid approach combining ultrasound and fluorescence could be an attractive solution, as proposed by several groups^24^,^25^,^26^. Such systems should be able to provide sensitive and specific molecular information through the addition of an optical module to the US systems already used by clinicians and the injection of an optical probe. Several prototypes have been developed and the technique has been successfully demonstrated *in vivo* on canine prostates^27^. However, in this context, a fluorescent probe with sufficient specificity is yet to be found.

We thus aimed to evaluate a fluorescent-labeled single chain variable fragment (scFv) of the D2B anti-PSMA antibody^21^ as a specific probe for the detection of prostate cancer by fluorescence imaging *in vivo*. The probe was evaluated using an orthotopic tumor mouse model and *in vivo* fluorescence tomography as the imaging method.

## Results

### Detection of PSMA in LNCaP-lucF cells by D2B and scFvD2B

**Figure 1:**
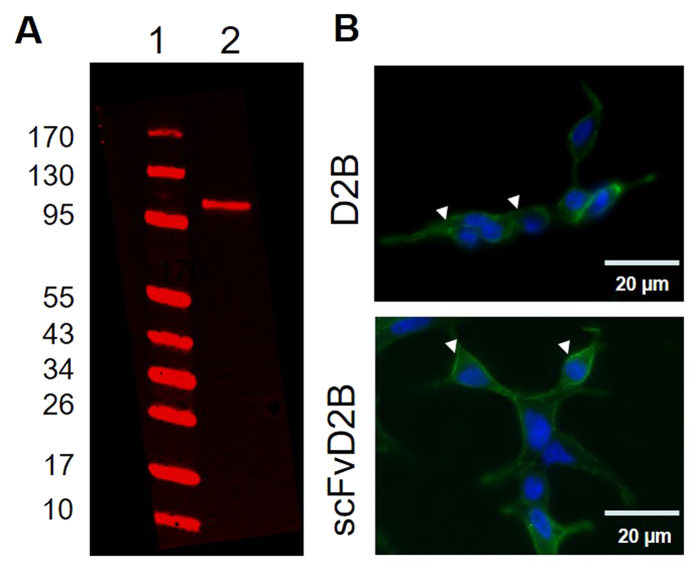
Evidence for PSMA expression and cell uptake by LNCaP-lucF cells (**A**) Western blot of LNCaP-lucF cell lysates using anti–PSMA D2B monoclonal antibody. Line 1: PageRuler marker, line 2: LNCaP-lucF cell lysate (20 μg proteins), (**B**) Immunocytochemistry on LNCaP-lucF living cells incubated with D2B or scFvD2B. Then D2B antibody was revealed with a goat fluorescent anti-mouse IgG and scFvD2B was revealed with an anti-His antibody, itself revealed with a goat fluorescent anti-mouse IgG. (n = 3 experiments)

The LNCaP-lucF cell line, expressing firefly luciferase constitutively, was obtained by stable transfection with pcDNA6.2-CMV-lucF and was first tested for PSMA expression by Western blot using the anti-PSMA D2B antibody (Fig. 1A). The whole D2B antibody and the scFvD2B format were further used for immunocytochemistry on living cells. D2B or scFvD2B (800 ng each/ 500 μL) were incubated for 1 hour with living LNCaP-lucF cells on coverslips at 37 °C. Then the D2B antibody was revealed with a goat fluorescent anti-mouse IgG and the scFvD2B was revealed with an anti-His antibody, itself revealed with a goat fluorescent anti-mouse IgG. A strong immunostaining signal was detected on the cell membranes and within the soma for both D2B and scFvD2B (Fig. 1B).

**Figure 2:**
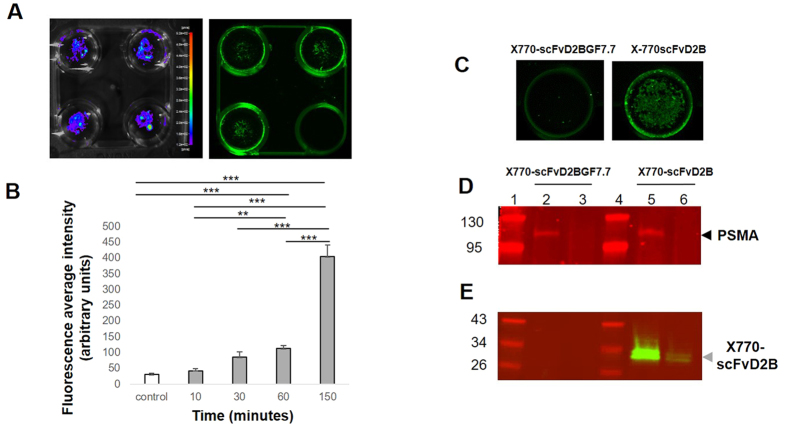
Internalization of the X770-scFvD2B by LNCaP-lucF cells. (**A**) LNCaP-lucF living cells were incubated with X770-scFvD2B probe and after washing scanned for fluorescence at 800 nm. As a control the lower right well did not contain X770-scFvD2B probe. BLI confirmed the presence of LNCaP-lucF living cells in each well. (**B**) Internalization of the X770-scFvD2B probe by LNCaP-lucF cells was followed by measuring the average fluorescence intensity at different times and compared to control wells without probes (n = 3; *** p < 0.01). (**C**) Fluorescence imaging of wells containing LNCaP-lucF cells incubated for 150 min with labeled scFvD2BGF7.7 or scFvD2B probes. (**D,E**) LNCaP-lucF incubated with labeled scFvD2BGF7.7 or scFvD2B probes were extracted for both cytosolic and membrane compartments. PSMA was detected by western blot using D2B antibody at 700 nm (**D**) and were mainly detected in membrane fraction (line 2 & 5) but not in cytosolic fraction (line 3 & 6). (**E**) X770-scFvD2B probe was detected at 800 nm in both membrane and cytosolic fractions (line 5 & 6) while X770-scFvD2BGF7.7 probe was not detected (line 2 & 3). PageRuler markers are lines 1 & 4. Each line corresponded to 119 000 LNCaP-lucF equivalent.

After labeling scFvD2B with Xenolight 770, other *in vitro* experiments were designed to monitor the binding properties of this new probe. LNCaP-lucF living cells were incubated with X770-scFvD2B probe (2 μg in 500 μL per wells) for up to 150 min. Cell distribution and viability were also followed by bioluminescence imaging (BLI) (Fig. 2A). At various time points and after a washing step cells were scanned for fluorescence and signals were compared with mock treated wells. As shown on Fig. 2A, a fluorescent signal remains within the cell after PBS washing. A significant increase of the average fluorescence signal within wells over the incubation time (from 10 to 150 min) was observed compared to the mock-treated controls (Fig. 2B).

To further investigate the link between probe internalization and scFv/PSMA interactions, a control scFv fragment called scFvD2BGF7.7 that cannot bind the PSMA antigen was synthesized. ScFvD2BGF7.7 was obtained by replacing the sequence of the D2B variable light chain with the sequence of the surrogate light chain VpreB^28^. The scFvD2BGF7.7 was further labeled with Xenolight 770 (X770-scFvD2B7.7). Living LNCaP-lucF cells were incubated (37 °C) with either X770-scFvD2B or X770-scFvD2BGF7.7 probes (2 μg/well) for 150 min and uptake was compared by measuring the fluorescence signal (Fig. 2C). In the wells containing LNCaP-lucF, no staining at 800 nm was detected with X770-scFvD2BGF7.7, whereas a fluorescent signal was observed with X770-scFvD2B (n = 6). Cells of each well were harvested, processed to isolate both membrane-bound and cytosolic proteins and submitted to Western blot using the anti PSMA D2B antibody. As expected, PSMA was detected at 700 nm mainly in the membrane fractions (Fig. 2D). A strong fluorescent 800 nm-signal (2 major bands) was found at about 30 kD in the membrane fraction, consistent with the molecular weight (MW) of the X770-labeled scFvD2B, and more slightly in the cytosolic fraction of the LNCaP-lucF cells incubated with the X770-scFvD2B probe (Fig. 2E). The observed MW suggested that scFvD2B and PSMA are not bound after extraction. No fluorescent signals were detected in samples from LNCaP-lucF cells incubated with the X770-scFvD2BGF7.7 probe. These results confirmed that probe internalization observed by LNCaP-lucF living cells is linked to interactions between PSMA and scFvD2B.

### Detection of X770-scFvD2B labeled LNCaP-lucF cells in the prostate using fluorescence molecular tomography (FMT)

**Figure 3:**
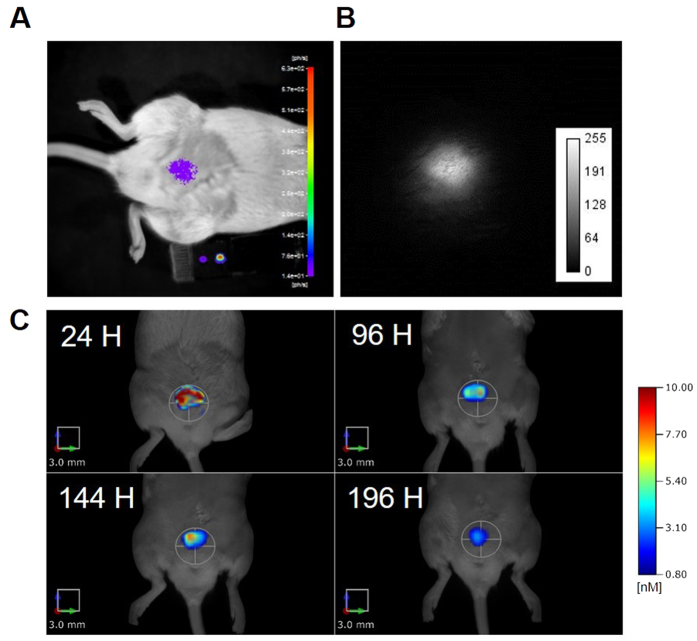
*In vivo* detection of X770-scFvD2B-labeled LNCaP-lucF cells injected into the prostate by BLI (**A**), FRI at 820 nm (**B**) and FMT at 790 nm (**C**), one day after surgical implantation of X770-scFvD2B-labeled LNCaP-lucF 10^6^ cells incubated for 2 hours with 10 μg X770-scFvD2B. (**C**) Time-course of the fluorescence signal (FMT) in the prostate 24, 96, 144 and 192 hours after surgery.

To test the feasibility of *in vivo* detection of fluorescent signals into the prostate, LNCaP-lucF cells were first incubated with X770-scFvD2B (10 μg/10^6^ cells) for 2 hours at room temperature to allow for internalization. Then, after 2 washes, cells (10^6^) were injected into the prostate by surgery (n = 3). Cell implantation and tumor growth were followed by BLI, fluorescent reflectance imaging (FRI) and FMT, daily. The X770-scFvD2B-labeled LNCaP-lucF cells, injected into prostate dorsal lobes, were detectable using BLI (Fig. 3A), FRI (Fig. 3B) and FMT (Fig. 3C). X770-scFvD2B-labeled LNCaP-lucF cells were detected by FMT from day one until day eight after surgery (Fig. 3C). These results showed that deep luciferase firefly activity could be detected *in vivo* in the prostate, and moreover the fluorescence signal from X770-scFvD2B-labeled cells could be followed daily in the prostate with FMT technology.

### *In vivo* targeting of prostate tumors by X770-scFvD2BGF7.7 versus X770-scFvD2B probes

**Figure 4:**
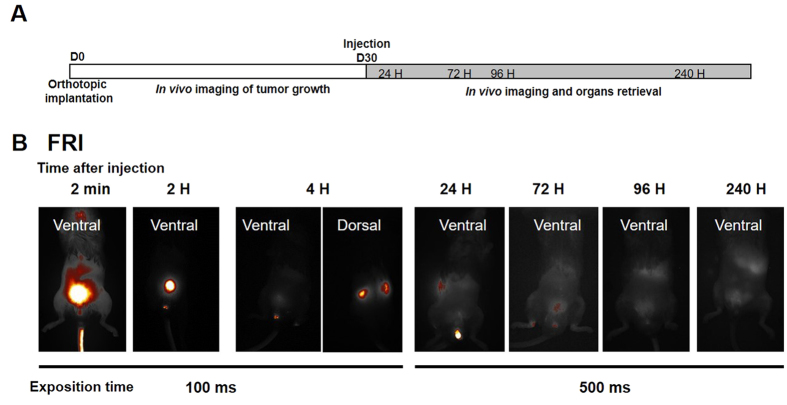
*In vivo* prostate tumor targeting by X770-scFvD2B. (**A**) Experiment flowchart. (**B**) Selected images by FRI of mice 30 days after LNCaP-lucF cell (0.5 × 10^6^ cells for each dorsal lobe) implantation. Mice were imaged by FRI at different times after X770-scFvD2B (80 μg) intravenous injection.

**Figure 5:**
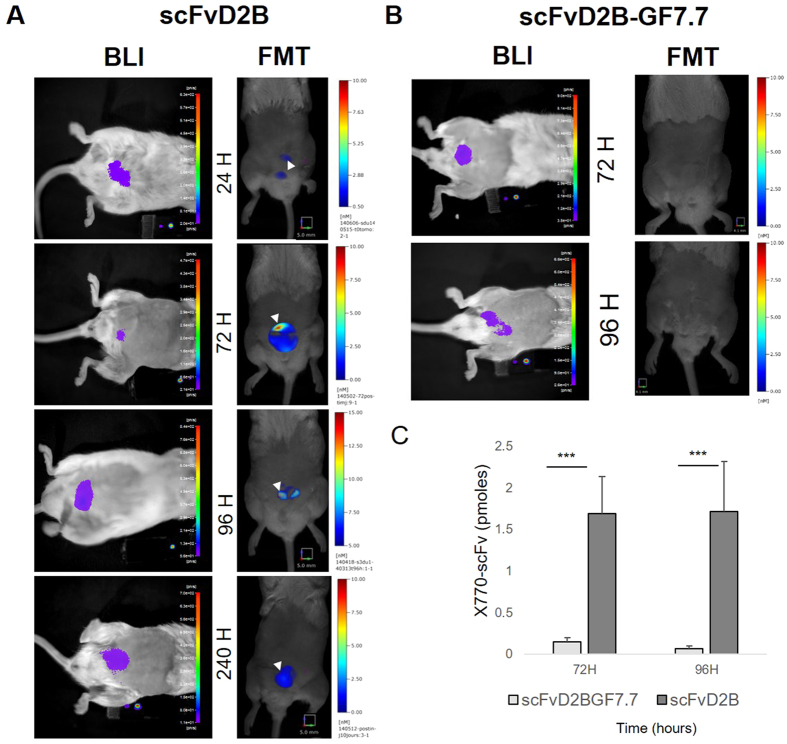
*In vivo* prostate tumor targeting by X770-scFvD2B (**A**) and X770-scFvD2BGF7.7 (**B**) probes. BLI of mice 30 days after LNCaP-lucF cell (0.5 × 10^6^ cells for each dorsal lobe) implantation at the time of intravenous 770-scFv probes (80 μg) injection. Each mouse was imaged by FMT at 24, 72, 96 or 240 hours after X770-scFvD2B (**A**) or X770-scFvD2BGF7.7 (**B**) injection. Quantification of the fluorescence signal recovered from FMT imaging at 72 and 96 h were plotted (**C**) allowing for comparison between X-770scFvD2B (n = 6 at 72 and 96 h) and X770-scFvD2BGF7.7-injected mice (n = 9 at 72 h and n = 4 at 96 h; *** p < 0.01).

The flowchart for the experimental procedure is summarized in Fig. 4A. All mice received LNCaP-lucF cells injected into the 2 dorsal prostate lobes (0.5 × 10^6^ cells per lobe). Tumor growth was checked by BLI (Fig. 5). Thirty days after intraprostatic cell inoculation of LNCaP-lucF, tumor growth was detected in all mice. X770-scFvD2B or X770-scFvD2BGF7.7 probes (80 μg) were injected in the tail vein and fluorescence distribution of the labeled probes was monitored by FRI and FMT at 24, 72, 96 and 240 hours. FRI revealed maximum fluorescence intensity at the injection site and in the bladder a few minutes after injection, then fluorescence slowly decreased but could still be detected in the kidneys at around 4 hours, and in the bladder and liver areas up to day 10 (Fig. 4B). By FRI, no fluorescent signal could be detected to the prostatic gland region. Detection and quantification of the fluorescence signal from X770-scFvD2B in the prostatic tumor (Fig. 5A, white arrows) was made possible by FMT analysis focused in the prostate region. The fluorescence signal was quantified 72 and 96 hours after injection. A very low fluorescence level was detected in the prostate region after X770-scFvD2BGF7.7 injection (Fig. 5B). Moreover, accumulation of the labeled scFvD2B with respect to the labeled control scFv increased 10-fold at 72 hours and 20-fold at 96 hours (Fig. 5C).

### *Ex vivo* analysis of the prostate

**Figure 6:**
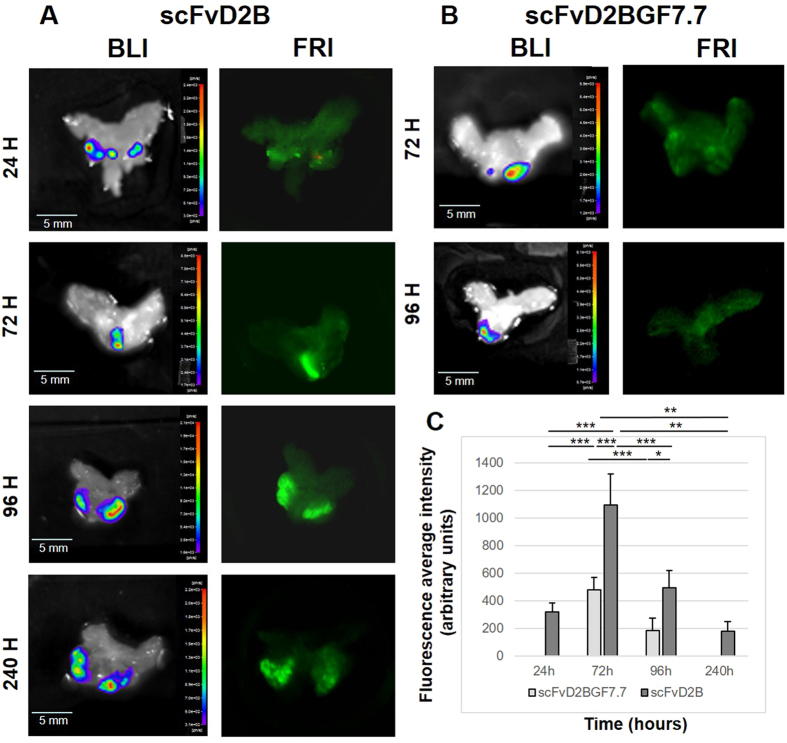
*Ex vivo* imaging of the prostate tumor after intravenous injection of X770-scFvD2B (A) or X770-scFvD2BGF7.7 (B) probes. The prostates were sequentially imaged by BLI and FRI at different times (**C**) Average intensity on whole prostate fluorescence after Odyssey scanning (n = 4 to 8 animals for each bar; ***p < 0.01).

Mice were sacrificed at 24, 72, 96 and 240 hours after intravenous administration of X770-scFvD2B and at 72 and 96 hours after X770-scFvD2BGF7.7 injection. Ten minutes before euthanasia, mice were injected with D-luciferin, thus excised prostates exhibited a BLI signal corresponding to lucF activity of LNCaP-lucF cells (Fig. 6A,B). Prostate from mice who received X770-scFvD2B injections exhibited a fluorescent signal overlapping the BLI signal (Fig. 6A). Animals injected with X770-scFvD2BGF7.7 showed low fluorescence retention within the tumor (Fig. 6B) that did not overlap with the BLI signal. X770-ScFvD2B fluorescence signals were quantified by scanning the prostate using an Odyssey scanner at 800 nm. As shown on Fig. 6C, the fluorescent signal in prostates from the X770-scFvD2B probe-injected mice peaked at 72 hours after injection and the fluorescence signal was significantly higher than in the prostates from control X770-scFvD2BGF7.7-injected mice.

### Immunohistochemical localization of X770-scFvD2B and X770-scFvD2BGF7.7 in the prostate cells

**Figure 7:**
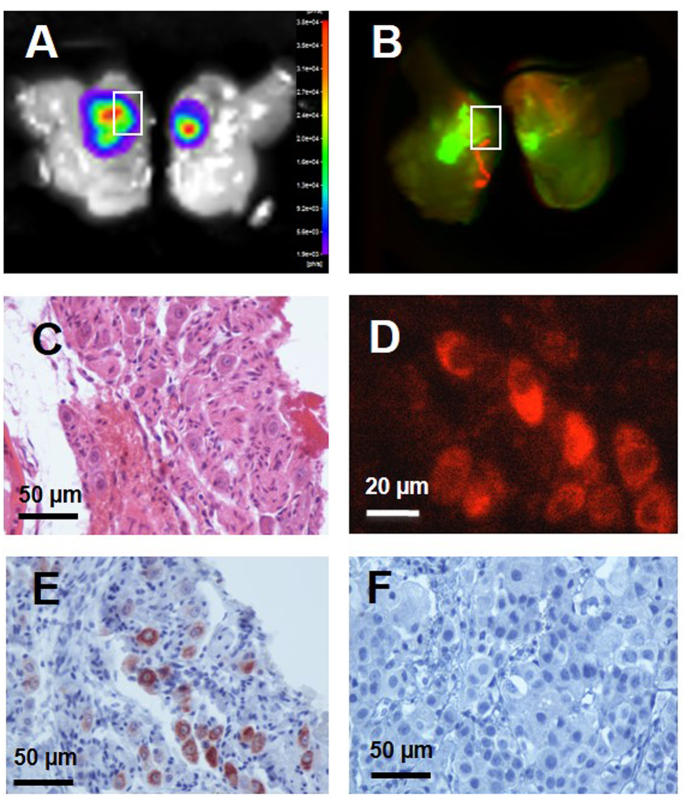
Detection of the X770-scFvD2B probe in the prostate 72 hours after *in vivo* injection. On bisected prostates (**A**) BLI revealed a prostate cancer tumor and (**B**) fluorescence scanning revealed the X770-scFvD2B probe. On the paraffin prostate slice (4 μm) tumor cells were revealed (**C**) by hematoxylin-eosin-safran staining and (**D**) X770-scFvD2B probe was detected by X770 fluorescence or (**E**) scFv His-tag detection using anti-His tag antibody (immunoperoxydase/DAB labeling). (**F**) No scFv His-tag was detected by immunohistochemistry after *in vivo* injection of the control fragment X770-scFvD2BGF7.7.

Histochemical and immunohistochemical detection were used to confirm the presence of prostatic carcinoma cells and to detect the X770-scFvD2B probe in the prostate (Fig. 7). Prostates were imaged by BLI (Fig. 7A) and FRI (Fig. 7B) to confirm colocalization of luminescent and fluorescent signals, then fixed and paraffin-embedded. HES staining revealed cancer cells and invasion of the prostatic parenchyma by the tumor cells (Fig. 7C). X770-scFvD2B was detected either directly by NIR microscopy due to the Xenolight 770 fluorophore (Fig. 7D) or by immunohistochemistry detection of the poly-histidine tag of the scFv fragment (Fig. 7E). No NIR fluorescence (data not shown) and no immunostaining was observed in mice injected with X770-scFvD2BGF7.7 (Fig. 7F).

## Discussion

In this study, we successfully applied, for the first time, noninvasive *in vivo* optical imaging to detect prostate cancer in an orthotopic mouse model using a fluorescent-labeled anti-PSMA antibody fragment scFvD2B. Specificity and binding properties of scFvD2B and its ability to internalize in PSMA-expressing cells both *in vitro* and *in vivo* has been previously described^21^. The present data showed that NIR fluorescent-labeled scFv, intravenously administrated reached the tumor and was internalized into PSMA-expressing cells. We further provided quantification of the fluorescence signal over time, corresponding to the presence of scFvD2B within the tumor. A control scFv that does not recognize PSMA did not label the malignant prostate tissue.

First, we developed an orthotopic mouse model of prostate cancer by intraprostatic inoculation of LNCaP cells genetically modified to follow tumor growth by BLI. Tumors occurred in 100% of operated mice and none of them developed metastases,^29^ mimicking the human clinical situation of a localized disease^29^.

As targeting moiety, we investigated the scFv format of the D2B antibody, described to have the same affinity for PSMA as the J591 whole antibody^21^ currently used in clinical trials^30^. The scFvD2B recognizes the extracellular domain of the PSMA protein. In the present work, we demonstrate that the scFvD2B is able to specifically detect PSMA on LNCaP cells *in vitro.* After intravenous injection of X770-scFvD2B in mice with PCa, a fluorescent signal was detected in the prostate, and this increased over 72 hours. Detection of the fluorescence was made possible by the use of FMT. FMT allows for 3D localization of deep fluorescent signals within the prostate, also allowing for absolute quantification. The localization of the X770-scFvD2B in PCa tumors was further confirmed on excised prostates by monitoring PCa tumor location by BLI and probe distribution by FRI. Our results showed that X770-scFvD2B binds to PSMA-positive cells and tumors with a strong staining, stable for at least 8 days after injection, whereas only a basal fluorescence was observed with the control antibody fragment scFvD2BGF7.7.

It has been shown that PSMA is internalized from the cell surface to the intracellular compartment *via* clathrin-dependent mechanisms^12^,^31^. Binding of antibodies or antibody fragments to the extracellular domain of PSMA has been shown to increase its rate of internalization 3-fold^12^ and PSMA internalization has been proposed as a vehicle to transport therapeutic molecules inside the cell^32^. In the current study, when LNCaP cells were treated with X770-scFvD2B, the fluorescent scFv fragment was found both in the membrane and in the cytosolic fraction, suggesting that the X770-scFvD2B probe is translocated into the cell through PSMA internalization. After intravenous injection of the X770-scFvD2B, the scFv fragment was detected in the tumor cells while injection of the control fragment scFvD2BGF7.7 did not accumulate in PCa cells. Overall, these observations suggest that internalization of the X770-scFvD2B probe is related to PSMA recognition and subsequent internalization. This mechanism may result in probe accumulation in PCa cells with maximal fluorescence intensity at 72 hours, but remaining detectable up to 7–8 days.

Today, the most widely-used strategies for molecular imaging of prostate cancer combine development of antibodies and small molecules (ligand or inhibitor)^33^,^34^ and radioactivity-based imaging. Currently the only FDA-approved antibody is 7E11 ProstaScint^® 15,16^ that binds the intracellular domain of PSMA. An alternative antibody J591 is currently being evaluated in a phase II clinical trial^35^. A small ligand for PSMA (BIND-014) is also currently being evaluated in a phase II trial,^36^ demonstrating the growing clinical interest in targeting PSMA. Recently, an anti-PSMA mAb, D2B, tagged with both^111^ In and IRDye 800CW was shown to be able to detect *in vivo* prostatic cancer cells in a subcutaneous xenograft model^17^. Regarding whole antibodies, scFv offered several advantages including lower immunogenicity. Its small size is also expected to facilitate better penetration within the tumor^37^ and may explain the rapid and strong signal observed in the current study using X770-scFvD2B probes. Furthermore, the peculiar feature of this scFv to show a high affinity in the nanomolar range (8.6 nM^21^), and the high level of internalization explain the persistence of this probe in the malignant prostate lesions of the treated mice.

Given the low rates of positive biopsies with current diagnosis techniques, new strategies are needed. MR-guided, MRI-US fusion-guided and contrast-enhanced US-guided biopsies are some of the currently proposed imaging solutions^38^. Targeting the primary tumor with optical probes, as reported in the current work using a high affinity and the very specific fluorescent-labeled anti-PSMA scFv, is, according to these preliminary preclinical results, a promising additional option. This method may help to specifically identify the tumor location and to guide transrectal sampling. This will require new imaging bi-functional (optical and US) devices, as reported recently^24^,^25^,^27^, and new algorithms for spatial co-localization of sonographic and optical data^26^. Thus, this approach consisting in *in vivo* staining of prostate cancer cells combined with an intraoperative optical imaging device could be used further to guide resection of malignant tumors providing a sensitive, real-time solution to reduce the risk of positive margins^18^.

## Materials and Methods

### Animals

All animal experiments were approved by the local ethical comity (CEEA 50) under agreement 50120196-A and all experiments were performed in accordance with European guidelines and regulations. Immunodeficient NOG (NOD/SCID/IL-2Rγ^null^) mice (6- to 10-weeks-old) were reared at the University of Bordeaux animal facilities. Animals were maintained in standard conditions under a 12-hours light/dark cycle with water and food provided *ad libitum.* Animals were anesthetized with 2% isoflurane (Belamont, Nicholas Piramal Limited, London, UK) in air for all surgical and imaging procedures.

For cell injection into the prostate, anesthetized mice were incised by a short abdominal longitudinal section of the skin and of the abdominal muscles. The seminal glands were grasped and pulled back outside the body. LNCaP-lucF cells (0.5 × 10^6^/10 μL per lobe) were injected in the dorsal prostate lobes. The seminal glands were repositioned within the abdomen and the incision was closed with sutures. Mice were imaged weekly by bioluminescence to monitor tumor growth starting three weeks after implantation. At selected times, labeled scFvD2B or scFvD2BGF7.7 (80 μg) was injected *via* the tail vein. Prostates were removed from euthanized animals, placed in cold PBS buffer on a glass slide and imaged.

### Production, purification of D2B, scFvD2B and scFvD2BGF7.7 and labeling

The monoclonal antibody D2B recognizing an external epitope of the PSMA antigen was produced by hybridoma technology and purified on protein G columns.^21^ The scFv format was synthesized by DNA technology from the purified mRNA of the D2B hybridoma as previously described^21^. The cDNA of scFv D2B was cloned in PHEN2 plasmid. The protein was produced in *E.Coli* HB2151 bacterial strain and purified on NiNTA columns (Qiagen, Courtaboeuf, France). The purification was checked by SDS-PAGE, binding capability and specificity were assessed by flow cytometry.

The control scFvD2BGF7.7, that is not able to bind PSMA antigen, was obtained replacing the sequence of the D2B variable light chain with the sequence of the surrogate light chain VpreB^28^.

The labeling reaction was made using Xenolight CF770 antibody labeling kit (Perkin Elmer, Waltham, MA, USA) on batches of 500 μg of each scFv. Labeled scFv were stored in the dark at 4 °C. Quantification of the labeling was made on 2 μg of each antibody fragments by Fluorescence Molecular Tomography (FMT 4000, PerkinElmer, Waltham, MA, USA) using system software (TrueQuant) calibrated with the fluorophore Xenolight770 CF (X770). Fluorescence labeling of scFvD2 and scfvD2BGF7.7 were 0.415 pmol/μg and 0.285 pmol/μg respectively. Fluorescence level of scfvD2BGF7.7 was thus corrected by a 1.45 factor for comparison with scFv.

### Cell line generation, culture and labeling

Prostate cancer cell line LNCaP (PSMA+) (ATCC^®^ CRL-1740^™^) was obtained from ATCC (Manassas, MD, USA). LNCaP cell line was grown in RPMI 1640 with Glutamax and 25 mM HEPES medium supplemented in 10% fetal bovine serum and 1% antibiotic-antimycotic mix (Life Technologies, Illkirch, France). LNCaP were stably transfected with pCDNA6.2-CMV-lucF using TransFast™ Transfection Reagent (Promega, Madison, WI, USA). The pCDNA6.2-CMV-lucF providing blasticidin resistance (10 μg/mL, Euromedex, Souffelweyersheim, France) was obtained by cloning lucF cDNA from a pGL3-basic vector (Promega) into pCDNA6.2 downstream the CMV promoter. Cells were incubated in humidified atmosphere 5% CO2/air at 37 °C.

For *in vitro* labeling of LNCaP-lucF with scFv, the cells were incubated with 2 μg/ 500μL of fragment at 37 °C, then washed (2 × 1 mL PBS (Life Technologies)). Samples were first scanned using Odyssey scanner (Li-Cor Biosciences, Nebraska, USA) then lysed using the Qproteome cells compartment kit (Qiagen).

### Western blotting

LNCaP-lucF (2 × 10^6^) cells were lysed using 400 μL of cold RIPA buffer (Sigma-Aldrich, Lyon, France) in the presence of a protease inhibitor cocktail (Fermentas, Illkirch, France). Samples (20 μg proteins) were analyzed by electrophoresis on 4–15% TGX stain-free gels (BioRad, Marne-la-Coquette, France) under denaturing conditions. Proteins were transferred to a nitrocellulose membrane using Transblot Turbo (Bio-Rad). PSMA was detected using mouse monoclonal D2B antibody (10 μg) and goat anti-mouse IRDye680-labeled (Li-Cor) as secondary antibody and analyzed on an Odyssey scanner at 700 nm. X770-scFvD2B or X770-scFvD2BGF7.7 were directly detected on the same membrane at 800 nm on an Odyssey scanner.

### Imaging

#### Bioluminescence imaging (BLI)

BLI was performed at Vivoptic (Bordeaux University) using a NightOWL II-LB 983 system (Berthold Technologies, Bad-Wildbad, Germany). Mice received an intra-peritoneal injection of D-luciferin (Promega, 2.9 mg in 100 μL PBS) and were sedated 7 min later. Bioluminescence images (1 min, 4 × 4 binning) and photographs (100 ms) were taken 10 min after luciferin injection. The bioluminescence signal was analyzed using Indigo software. Prostates, removed from euthanized animals immediately after *in vivo* BLI, were placed in cold PBS on a glass slide and imaged (1 min). LNCaP-lucF cells in PBS in 12-well culture plates were imaged (1 min) by BLI immediately after adding D-luciferin (6.10^−4 ^M in 200 μL PBS).

#### Fluorescence Molecular Tomography (FMT)

Anesthetized mice were placed in a FMT 4000 apparatus (Perkin Elmer Inc., Boston, MA, USA). Scanning was performed using the 790EX channel on the abdominal part of mice. The images were reconstructed using the TrueQuant software. The same ROI were drawn to encompass the prostate region in each animal. The total amount of fluorescence (pmoles) was calculated relative to calibration with Xenolight770 CF.

#### Fluorescence reflectance imaging (FRI)

Animals were imaged using the real-time intraoperative camera system Fluobeam^®^ (Fluoptics, Grenoble, France) at a spectral window of excitation at 780 nm and emission at 820 nm. Prostates with tumor were placed into 12-well plates in cold PBS and scanned at 800 nm on Odyssey scanner.

#### Immunocytochemistry on living cells

LNCaP-lucF were cultured on coverslips, were incubated with D2B antibody or scFvD2B (800 ng/ 500 μL) in RPMI complete medium for 1 hour at 37 °C and fixed 10 min in 4% paraformaldehyde. Using D2B antibody, cells were incubated with goat Alexa-Fluor 488 anti-mouse IgG (Invitrogen, 1/400 in PBS 1% BSA) with DAPI (1/5000) for 1 hour and finally mounted in Fluoromount medium (Deltamicroscopies, Mauressac, France). Using scFvD2B, cells were incubated with mouse anti-His monoclonal antibody (HIS-H8, Pierce, Rockford, IL, USA) 1/500 in PBS 1% (w/v) BSA) for 1 hour at 20 °C, with Alexa-Fluor 488 anti-mouse IgG (1/500 in PBS) with DAPI (1/5000) for 1 hour and finally mounted. Observations were performed on a Leica DM 5500 microscope (Wetzlar, Germany).

#### Histochemistry and Immunohistochemistry

Prostates with tumors were fixed overnight at 4 °C with 4% paraformaldehyde. Then the tissues were dehydrated and embedded in paraffin. Sections (4 μm) were used for hematoxylin/eosin/safran (HES), scFvD2B and scFvD2BGF7.7 staining after dewaxing and rehydration in alcohol. The scFv fragments (scFvD2B and scFvD2BGF7.7) were detected using anti-Histidine antibody HIS-H8 (Pierce). The DAKO Envision FLEX detection system included pretreatment with DAKO PT Link (pre-treatment module) and target retrieval solution, pH 6 (K800521) 97 °C for 20 min. All steps of immunostaining were done in an automated immunostainer (Autostainer Plus, DAKO, Les Ulis, France). Inhibition of endogenous peroxidases (H_2_O_2_ 3% in water, 10 min) was followed by incubation of the primary antibody (1/250, 45 min). Sections were incubated with HRP conjugated Envision (DAKO, 20 °C, 20 min) and revealed with DAB. Images were obtained on Nikon Eclipse 50i using NIS-element F software. Fluorescence from X770 was also detected on the processing slices using Leica DM 5500 microscope fitted with pE-100 (Ex 770 nm) Cool LED and a indocyanine (775/845 nm) filter (Leica Microsystems).

#### Statistical analysis

Statistical analysis included the ANOVA test for independent data and a post hoc test of Bonferroni. In all figures, error bars indicate standard deviations. *** for p < 0.01; ** for p < 0.05; * for p < 0.1.

## Additional Information

**How to cite this article**: Mazzocco, C. *et al. In vivo* imaging of prostate cancer using an anti-PSMA scFv fragment as a probe. *Sci. Rep.* **6**, 23314; doi: 10.1038/srep23314 (2016).
